# Optimal Pathways to Lung Cancer Screening in Primary Care Settings: A Scoping Review

**DOI:** 10.3390/curroncol32010008

**Published:** 2024-12-26

**Authors:** Emmanouil K. Symvoulakis, Izolde Bouloukaki, Antonios Christodoulakis, Antonia Aravantinou-Karlatou, Ioanna Tsiligianni

**Affiliations:** 1 Department of Social Medicine, School of Medicine, University of Crete, 71500 Heraklion, Greece; i.bouloukaki@uoc.gr (I.B.); christodoulakisa@uoc.gr (A.C.); medp2012149@med.uoc.gr (A.A.-K.); i.tsiligianni@uoc.gr (I.T.); 2 Department of Nursing, School of Health Sciences, Hellenic Mediterranean University, 71410 Heraklion, Greece

**Keywords:** lung cancer, screening, primary care, review

## Abstract

Lung cancer is the leading cause of cancer-related deaths worldwide, and delayed detection contributes to poor outcomes. Primary care plays a crucial role in early diagnosis, but detecting lung cancer early remains challenging for general practitioners (GPs). Therefore, the aim of this scoping review was to identify optimal strategies and pathways for lung cancer screening (LCS) in primary care settings globally. We conducted a scoping review by searching PubMed, Scopus, and the Cochrane Library for relevant studies published in the past 10 years. Our keywords included “lung cancer”, “primary care”, “early detection”, “screening”, “best practices”, and “pathways”. We included randomized controlled trials, cross-sectional studies, and cohort studies focused on lung cancer screening in primary care. We extracted data on study characteristics, screening pathways, and key findings. We identified 18 studies that met our inclusion criteria. Important strategies for LCS included the use of shared decision-making tools, electronic health record (HER) prompts, risk prediction models, community outreach, and integration with smoking cessation programs. Barriers to implementation included the lack of provider familiarity with guidelines, time constraints, and patient factors. Healthcare professionals and policy makers in primary care settings can leverage this information to integrate the most effective screening strategies into their care, thus enhancing early detection rates and subsequently reducing global lung cancer morbidity and mortality.

## 1. Introduction

Lung cancer remains one of the leading causes of cancer-related morbidity and mortality worldwide, with nearly 2.5 million new cases and over 1.8 million deaths globally [1]. While there have been notable advancements in managing early- and advanced-stage lung cancer, the level of improved outcomes achieved in other cancer types over the past four decades has not been attained [2]. This is because most lung cancers are often detected at later stages when curative treatment is no longer an option [3]. As a result, the 5-year survival rate for lung cancer is typically less than 20% in the majority of countries [4] and seems to be influenced by treatment, healthcare systems, and the prevalence of comorbidities [5], as well as the late identification and delayed diagnosis of the disease [5]. Therefore, early detection is crucial to improve treatment effectiveness and disease outcomes and reduce healthcare costs [6].

General practitioners (GPs) play a pivotal role in identifying lung cancer early and enabling prompt diagnosis and treatment, since they are typically the first point of contact to the healthcare system for most patients with lung cancer [7,8,9,10]. Given their role, GPs are in a favorable position to approach high-risk individuals, initiate conversations about screening, and offer continuous support during the screening process [11]. By utilizing interventions, such as shared decision-making tools integrated into electronic health records, GPs can enhance participation in lung cancer screening and adherence to screening guidelines [12]. Additionally, GPs can encourage behavioral changes, such as quitting smoking, which supports lung cancer screening by targeting a major modifiable risk factor [13].

Nevertheless, the early detection of lung cancer has posed a significant challenge for GPs due to the similarity of its symptoms to less severe respiratory conditions, resulting in misdiagnosis and subsequent delays in treatment [14]. Studies indicate that around one-third of individuals diagnosed with lung cancer have made three or more visits to their GP with symptoms that can be linked to their cancer before receiving a diagnosis [15], while one-third of lung cancer cases are detected when patients seek emergency medical care. To address these issues, various strategies/interventions have been developed and implemented to enable the timely detection of lung cancer. An important strategy is the implementation of lung cancer screening programs focusing on individuals at high risk, primarily smokers and ex-smokers [16]. Several randomized controlled trials have demonstrated that the mortality rates associated with lung cancer have been effectively reduced with the use of low-dose computed tomography (LDCT) [17,18]. For individuals aged 50–80 with a smoking history of 20 pack-years, whether they are current smokers or have quit within the last 15 years, the US Preventive Services Task Force (USPSTF) recommends getting an annual lung cancer screening using LDCT [19]. In a recent guideline update, the American Cancer Society revised the criteria for years since quitting to include former smokers who have surpassed 15 years since quitting [20]. In Europe, the European Commission has suggested the need for lung cancer screening to all 27 member states as part of its Europe’s Beating Cancer Plan [21]. These guidelines and interventions have played an important role in improving the screening process of lung cancer in primary care settings; however, it is evident that there is still potential for improvement at a global scale [22].

Improving lung cancer screening in primary care requires a multifaceted approach, a healthcare services re-design, and the identification of more efficient diagnostic pathways, incorporating patient engagement tools, providers education, and targeted outreach to high-risk groups. [23,24,25]. There is also evidence that linking smoking cessation programs with lung cancer screening efforts might yield greater advantages. [11,16]. By incorporating all these preventative measures and interventions into clinical practice, GPs can provide a comprehensive approach to lung cancer prevention. This would involve addressing both the immediate need for screening for eligible individuals and promoting long-term behavioral changes, such as quitting smoking [11,26,27]. Therefore, the aim of this scoping review was to identify the most optimal screening strategies and/or pathways for lung cancer screening in primary care settings, worldwide. Healthcare professionals and policy makers in primary care settings can leverage this information to integrate the most effective screening strategies into their care, thus enhancing early detection rates and subsequently reducing global lung cancer morbidity and mortality.

## 2. Materials and Methods

### 2.1. Methodological Approach

In accordance with the JBI guidelines for conducting scoping reviews [28], this scoping review was carried out, and the results were documented using the PRISMA-ScR checklist [29]. Studies had to fulfill the following criteria to be considered for inclusion in this review: (1) being peer-reviewed articles published in English, (2) focusing on lung cancer, (3) investigating optimal strategies and pathways for lung cancer screening, (4) encompassing randomized controlled trials, cross-sectional studies, cohort studies, (5) studies including primary care settings, and (6) being published in the past ten years. The exclusion criteria involved the exclusion of non-peer-reviewed articles, editorials, opinion articles, and studies that addressed different topics and non-optimal strategies and pathways for lung cancer screening. The process of selecting studies consisted of two stages: an initial screening of titles and abstracts, followed by a comprehensive review of full-text articles. Two reviewers performed the screening process individually. To resolve discrepancies between the reviewers, discussion was employed, and a third reviewer was included to achieve a consensus. In order to ensure transparency and reproducibility, we utilized a PRISMA flow diagram to record the selection process.

### 2.2. Information Sources and Study Selection

Our search strategy involved a comprehensive approach to ensure the identification of relevant literature. Electronic databases were used to guarantee comprehensive coverage of medical and scientific journals, PubMed, Scopus, and the Cochrane Library from August to September 2024. A mixture of keywords and Medical Subject Headings (MeSH) associated with optimal strategies and pathways for lung cancer screening in primary care was used in the search. Consequently, we implemented the use of the following keyword combinations and Boolean operators (AND, OR) in the databases: “lung cancer”, “primary care”, “early detection”, “screening”, “best practices”, “strategies”, and “pathways”. Primary outcomes were number of referrals for lung cancer screening (LCS), number of consultations for LCS, number of patients screened for lung cancer, and number of patients diagnosed with lung cancer.

### 2.3. Data Synthesis and Analysis

In this scoping review, we analyzed data regarding optimal strategies and pathways for lung cancer screening in primary care. The procedure consisted of obtaining information regarding the study design, lung cancer screening pathways in primary care, full texts, and the relevant results from the included studies. Two reviewers employed a standardized data extraction form for data collection. Following the elimination of duplicate entries, a meticulous appraisal process was conducted, in which two independent reviewers evaluated each extraction form and deliberated on any discrepancies. The extracted data have been enriched with supplementary information, encompassing the author, year, study design, country, place, and participants of the studies. Furthermore, information was supplied pertaining to the size of the sample. The information comprised the study design and the primary findings concerning pathways for lung screening. The data extraction was carried out by two independent reviewers in order to ensure both accuracy and consistency. The findings were synthesized, then categorized according to the review outcomes.

## 3. Results

### 3.1. Screening and Procedure

**Figure 1:**
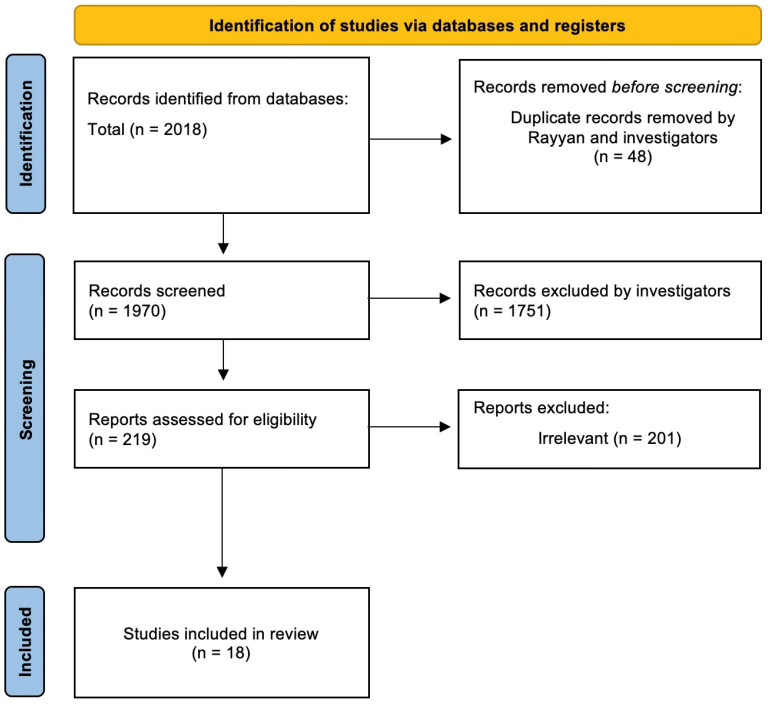
PRISMA flow diagram for this scoping review.

The database search for this scoping review produced a total of 2018 studies. After the first screening and removal of duplicates, a total of 1970 studies underwent screening, which was conducted by assessing their titles. Following that, a total of 219 titles were identified that satisfied the inclusion criteria and were subsequently selected for further evaluation, with a primary focus on their abstracts. Afterwards, a second evaluation was performed on abstracts that met the criteria, and their full texts were obtained for further screening. Nonetheless, 201 studies were disregarded due to their non-compliance with the inclusion/exclusion criteria specified in the methodology. Consequently, a total of 18 full-text studies were ultimately incorporated in this scoping review. The PRISMA flow diagram in Figure 1 shows the process for the literature search.

### 3.2. Overview Characteristics of the Included Studies

The overview characteristics of the 18 included studies [30,31,32,33,34,35,36,37,38,39,40,41,42,43,44,45,46,47] have been outlined in Table 1. The majority of the studies were categorized as of an interventional, pre–post study design. The analysis included 11 studies from the USA [30,31,32,35,36,37,40,41,45,46,47], 3 studies from the UK [34,38,39], 1 study from Korea [44], 1 study from Brazil [33], 1 study from Canada [42], and 1 study from Poland [43]. Sample sizes ranged between 50 and 969,351. The studies were published from January 2017 to December 2023.

### 3.3. Description of Interventions Implemented to Facilitate the Uptake of LCS

Seven interventions were conducted to improve decision-making for LCS, with a specific focus on shared decision-making (SDM) sessions (Table 2) [31,32,35,37,40,42,47]. Ten interventions included the use of decision aids, educational tools, and outreach materials [30,31,32,36,40,42,43,44,46,47]. Most interventions also emphasized the utilization of electronic health records as a means to reach eligible patients (Table 2) [30,31,32,35,36,38,39,40,41,42,43,44,46,47]. Five of these interventions encouraged patients to directly seek risk assessments [32,36,42,46,47], while the others involved clinical prompts and reminders for healthcare providers in primary care settings [30,31,32,35,40]. Three studies outlined community-focused interventions, which involved initiatives like community campaigns or the utilization of mobile computed tomography scanners [33,34,38]. Five interventions were found to include a smoking cessation component, such as referring smokers to hotlines or specialized clinics for nicotine dependence [33,34,35,37,47].

The Bach’s model predictors include (I) age; (II) gender; (III) asbestos exposure; (IV) smoking intensity (cigarettes per day); (V) smoking duration; and (VI) quit time in former smokers.

### 3.4. Efficiency and Levels of Lung Cancer Screening

Most interventions outlined in this review improved the accessibility of LDCT by increasing awareness of LCS services among at-risk populations (Table 2). Three studies utilized community campaigns or mobile computed tomography scanners [33,34,38] to identify ways of raising awareness of LCS in ways that encourage people at higher risk to come for screening. However, the response screening rate was not as high as anticipated, possibly because of the diverse groups targeted.

The use of electronic health records to reach eligible patients offers a pathway to implementing LCS effectively in primary care (Table 2) [30,31,32,35,36,38,39,40,41,42,43,44,46,47]. Identifying patients eligible for LCS but who had not received LDCT screening through the health electronic record system and targeting them was an effective intervention that greatly boosted LCS [36]. This is significant for patients in socioeconomically challenged areas, and two studies have highlighted the importance of adapting interventions to the sociocultural characteristics of the target population to increase its acceptance [34,45]. Furthermore, lung cancer risk assessment tools like ALIGNED [39] can calculate scores without direct patient interaction and appear to be more effective than the ever-smoked criteria from earlier guidelines. These tools could serve as an initial approach to lung cancer screening by providing personalized data to help individuals make better-informed decisions.

### 3.5. Detection of Lung Cancer

Ten of the eighteen included studies reported directly on patient outcomes relating to LDCT findings (mainly Lung-RADS scores) [30,32,46] and the detection of LC (Table 2) [33,35,37,43,44,45]. Taking into account that higher Lung-RADS scores correspond to increased likelihood of malignancy [48], the prevalence of Lung-RADS scores 4B/4X was significantly higher (more than double) than anticipated in one study [32].

### 3.6. Engagement of GPs with Lung Cancer Early Detection and Referral

In five studies, the involvement of healthcare professionals in early detection and referral processes for lung cancer improved LCS (Table 2) [30,31,32,35,36]. It is worth noting that the implementation of a smoking history protocol by Brenner et al. [31] raised the completion rate of smoking histories from 22% before the test to 47% post-test. Several studies have found that involving both the clinician and the patient in decision-making can enhance clinician engagement in the referral process by improving the documentation of the decision-making steps [31,32,35,37,40,42,47].

## 4. Discussion

This scoping review aimed to identify strategies/interventions in which primary care could improve the implementation of LCS among high-risk populations. Our findings suggest that tailored interventions to primary care, including the use of shared decision-making tools, prompts within electronic health records, increasing awareness of LCS services among at-risk populations, and the integration of smoking cessation interventions with LCS could be utilized and improve the uptake of LCS. Each of these strategies contributes in its own unique way to the early detection of lung cancer in high-risk individuals and thus could improve the disease outcomes.

One important finding of our review was the positive effects of using SDM tools to improve LCS uptake. By incorporating SDM, GPs could have structured and productive discussions about screening, address patient concerns, and correct any misconceptions. According to Kukhareva et al., these tools have a significant impact on increasing patient engagement in screening decisions, leading to a higher participation rate in LDCT among high-risk individuals [40]. SDM tools have the potential to improve communication, simplifying the process for providers to manage complex medical information and personalize care for each patient. This systematic approach could be particularly valuable in tackling common challenges, such as patient anxiety concerning screening results or their lack of knowledge about the benefits of LCS.

Another finding in this review was that three studies [10,14,15] included prediction models that performed better than previous screening programs based on age and pack-years. However, to integrate the current lung cancer risk prediction models into regular primary care clinical practice, it would be necessary to directly interact with patients, since EHRs may not contain the detailed information needed about individual risk factors [49,50,51,52,53]. Previous studies have shown that there are disparities in the completeness and quality of smoking records in EHRs [34,38,52,54,55,56,57]. These studies specifically emphasize how deprivation, age, and ethnicity inequalities can impact the accuracy and reliability of these records [34,38,52,54,55,56,57]. Indeed, the accuracy of smoking data in primary care EHRs has been found to be low or moderate, with significant missing data [50,53]. Moreover, smoking information is often found in various clinical notes and structured forms, but it lacks standardization, which poses challenges for clinical decision support tools and healthcare providers to identify a smoking history accurately from the information available in the EHR [58]. Another major challenge is the lack of data regarding the usage rates of electronic cigarettes (e-cigarettes) in EHRs [59,60]. This is important, as accurate, complete, and consistent e-cigarette use status documentation in EHRs is crucial for enabling the investigation of the long-term health effects of e-cigarettes [59,60]. Although conclusive evidence is currently unavailable, the existing data suggest a link between e-cigarettes and a higher risk of lung cancer [61]. Therefore, given the rising prevalence of e-cigarette use, particularly among young people, an update of the current lung cancer screening protocols is expected [62,63]. For example, one possible strategy to identify a smoking history accurately [12] could be the combination of smoking information from clinic notes with structured smoking data (i.e., validated questionnaires) and subsequently the evaluation of smoking history in determining eligibility for LCS [41]. This method has the potential to simplify the process of identifying patients who are eligible for LCS and can be a technological basis for developing a clinical decision support tool in the future by leveraging the newest AI technologies [64].

The utilization of established risk factors obtained from EHRs could facilitate the development of decision support tools, including interactive online software applications and algorithms designed to categorize individuals into different risk groups [65,66]. There have been several identified risk factors that are used to predict the likelihood of developing lung cancer. Previous studies on LCS have primarily focused on two key factors, namely age and smoking history, as the main criteria to identify high-risk populations [18,67,68,69,70], with screening rates for eligible patients ranging from 5% to 18% [71]. Over the past few years, several models have been created to predict the risk of lung cancer, and they have been found to be highly effective in identifying individuals with a high risk [72,73]. These models typically incorporate several common risk factors, such as detailed information on sex, race/ethnicity, chronic obstructive pulmonary disease, smoking habits (including duration, quit time, and intensity), a family history of lung cancer, and lifetime exposure to radon and asbestos [72,73]. Previous research has shown that using a risk prediction model-based approach in lung cancer screening leads to a significant decrease in lung cancer mortality [72,73].

Another key finding from the current review relates to the potential positive impact of smoking cessation interventions in lung cancer screening participants [4,5,6,8,18]. Indeed, smoking cessation counseling could be provided concurrently with LCS [74]. Within this particular context, lung cancer screening offers a prime opportunity to address smoking cessation, given that participants in the screening program are typically followed up for an extended period of time and are likely to prioritize their health more than those who are eligible but do not participate. According to previous research, including a smoking cessation intervention in the LCS program can effectively encourage screening participants to quit smoking, and an increase in smoking cessation rates plays a vital role in improving the cost-effectiveness of lung cancer screening, especially in national screening programs [75].

Our review also emphasized the role of LDCT as a promising method for large-scale screening aimed at the early diagnosis of lung cancer [76]. Despite the approval of LDCT screening by the US Preventive Services Task Force (USPSTF) in 2013, uptake has been reported to be as low as 2–5% [77,78,79,80]. Low initial LCS rates have been linked to patients’, providers’, and systems’ factors [81]. For example, a considerable number of individuals who eventually develop lung cancer were not eligible for LDCT screening based on age and/or smoking history [24,78]. Moreover, LDCT is not readily accessible/available to all primary care settings [82]. In this case, chest radiography is the initial radiological diagnostic method commonly used by GPs in primary care settings [83]. While the sensitivity of chest radiography is limited, identifying lung cancer in only 77–80% of cases in the year before diagnosis [84], the use of computer-aided diagnosis and AI-based software could assist in the detection of clinically significant lung nodules on chest radiography [85]. However, early-stage lung cancer often lacks noticeable symptoms, posing difficulties in achieving an early diagnosis [86]. As a result, even if screening becomes widely used, the majority of patients will still receive a diagnosis after they start experiencing symptoms [2]. Furthermore, in order to achieve an early diagnosis, it is crucial for GPs to uphold a suitable level of suspicion and preparedness to investigate patients at high risk or those exhibiting persistent symptoms [27]. To achieve effective lung cancer screening in primary care settings, it is important to adopt a multifaceted approach that prioritizes risk assessment, patient education, interdisciplinary teamwork, and ongoing evaluation [26]. GPs should actively participate in a healthcare services re-design and identify more efficient diagnostic pathways, integrating decision support tools into their consultations, drawing from validated lung cancer risk models [87].

The incorporation of electronic prompts into EHRs utilized in five articles [1,2,3,6,11] could be an important approach for LCS in primary care settings to facilitate shared decision-making and encourage LCS uptake. Web-based technologies may hold promise, as there is now software accessible that permits patients attending primary care appointments to receive and answer electronic questionnaires before their consultation. Pre-consultation software can address the issue of inconsistent physician recommendations for high-risk patients to undergo LDCT [88] by improving the screening process with a more standardized approach. Furthermore using a lung cancer screening decision aid [89], included in most of the articles in this review, can be a useful tool for PCPs to effectively communicate the complex information regarding the advantages and disadvantages of screening. On the other hand, a different approach is to invite the entire eligible age group by sending letters and offering lung health check-ups to individuals who have a history of smoking [68], an approach highlighted in three of the studies included [4,5,9]. This approach lowers the chances of excluding eligible individuals because of missing or inaccurate primary care records, but it does come at a higher cost due to the increased number of letters that need to be mailed. Additionally, it is not known whether receiving an invitation brings harm, such as undue anxiety to individuals that are finally not eligible for screening. Nevertheless, and although the most effective approaches for identifying potentially eligible asymptomatic patients in primary care remain uncertain, the requirements for LCS should involve utilizing EHRs to identify eligible patients, attending a shared decision-making visit where the potential advantages and drawbacks of screening are discussed, conducting an LDCT scan with specific parameters for screening, discussing the results with the patient, ensuring a multidisciplinary follow-up of the screening results, and offering smoking cessation counseling. However, it is unclear whether all the required components for lung cancer screening can be implemented, particularly in [90,91] ethnic minorities, individuals with low socioeconomic status, and those with limited access to healthcare [92,93,94]. Therefore, based on the aforementioned evidence, combined with the emphasis placed by medical organizations on the clinical utility of screening, there is a need to prioritize an equitable implementation of LCS programs in order to effectively reduce the morbidity and mortality rates of lung cancer [95,96,97,98].

This review further underscored the crucial role of primary care in providing the initial access point to eligible individuals for LCS [99]. LCS is typically implemented through a decentralized model, relying on GPs to contribute to the program’s success by promoting the identification and invitation of high-risk patients, as well as fostering equitable and informed participation [100,101]. Furthermore, GPs could contribute to LCS programs by facilitating decision-making visits, ensuring annual follow-ups, determining the appropriate next steps for positive screening scans, and offering smoking cessation counseling [102]. However, GPs often face significant obstacles when trying to implement LCS. These barriers include a lack of familiarity with screening guidelines, challenges in identifying eligible patients, insufficient training in SDM, limited time for SDM discussions, competing clinical priorities, and a need for additional support in managing follow-up testing or abnormal results [81,82,103]. In addition, GPs are faced with a broader range of responsibilities, which further hinders their ability to provide effective LCS [104]. This includes an increased demand for documentation and limited time available for outpatient care [104]. On the other hand, patients also encounter obstacles [53,55]. These include a lack of understanding about the purpose of LCS, anxiety surrounding a potential cancer diagnosis, limited access to healthcare services, instances of smoking-related discrimination, and a lack of trust in the healthcare system [53,55]. These barriers disproportionately affect underrepresented minorities, individuals with low socioeconomic status, and those residing in rural areas [55]. Recent research indicates that the ongoing pandemic has exacerbated these challenges, resulting in neglected chronic illnesses and an uptick in comorbidity among patients [105,106]. Healthcare facilities have been compelled to innovate and adapt their screening procedures in order to maintain essential services while minimizing the risk of virus transmission. Some of these adaptations include the utilization of telemedicine consultations for initial assessments, the development of risk stratification tools to prioritize high-risk patients for in-person screenings, and the implementation of stricter infection control measures during necessary visits [105,106].

### Limitations

This review has a few limitations that may impact the interpretation of the findings and the generalizability of the results. Firstly, the heterogeneity in study designs and screening approaches among the included studies introduces variability in screening outcomes, making it difficult to directly compare interventions or draw universal conclusions. Moreover, the variability in primary care settings, screening eligibility criteria, and population characteristics across different regions further complicates direct comparisons. Secondly, there may be a publication bias that has influenced the review findings. Studies with positive or significant outcomes are more likely to be published, while those with neutral or negative findings may be underrepresented. Additionally, some studies had limited sample sizes or short follow-up periods, which restricts the ability to assess long-term effectiveness and patient adherence to lung cancer screening protocols in primary care settings. Lastly, it is important to note that the articles included in our study may not include all the relevant patient populations, especially those facing barriers to accessing care such as socioeconomically disadvantaged groups or those with limited healthcare access. These limitations underscore the need for further research to evaluate standardized, scalable interventions and assess their effectiveness across diverse healthcare settings and patient demographics.

## 5. Conclusions

In conclusion, our findings highlight the crucial role that primary care plays in promoting lung cancer screening and early detection. By incorporating tools for SDM, EHR prompts, and community outreach, GPs could improve the uptake of LDCT screening and more efficiently identify high-risk individuals. Moreover, including LCS in smoking cessation programs has a particularly positive impact, as it could reduce long-term lung cancer risks and increase patient engagement. However, there are still challenges that need to be addressed, such as limited provider familiarity with screening guidelines, which hinder the optimal adoption of these practices. To overcome these barriers, a targeted training in SDM, the optimization of EHR systems, and expanded community engagement initiatives are needed to improve accessibility and effectiveness. By implementing these approaches, GPs could achieve more positive outcomes in lung cancer screening, emphasizing the importance of tailored primary care interventions in cancer prevention efforts.

## Figures and Tables

**Table 1:** Characteristics of included studies.

Author/Year (Ref.)	Study Design	Country	Setting/Participants	Eligibility Criteria [Age (y); Smoking Exposure (py); Quit Duration (y)]
Azubuike et al., 2020 [30]	Interventional, pre–post study design	USA	Family medicine clinic N = 27	USPSTF 2013 criteria [55–74; ≥30; ≤15]
Brenner et al., 2018 [31]	Interventional, pre–post study design	USA	Academic primary care clinic N = 2349	[55–80]
Chiarantano et al., 2022 [33]	Interventional, pre–post study design	Brazil	Community N = 233	National Lung Screening Trial criteria [55–74; ≥30; ≤15]
Colamonici et al., 2023 [32]	Interventional, pre–post study design	USA	Primary care center N = 341	[50–74; ≥20; ≤15]
Crosbie et al., 2022 [34]	Interventional, RCT	UK	Primary care center N = 89,917	USPSTF 2013 criteria [55–74; ≥30; ≤15] or PLCOm2012 (6-year risk ≥1.51%) or LLP criteria (5-year risk ≥5%)
Currier et al., 2022 [35]	Interventional, pre–post study design	USA	Rural primary care and community hospital N = 567	USPSTF 2013 criteria and CMS screening guidelines [55–74; ≥30]
DiCarlo et al., 2022 [36]	RCT	USA	Primary care practices N= 2376	USPSTF, CMS, and National Comprehensive Cancer Center Network guidelines [50–74; ever-smokers]
Fagan et al., 2020 [37]	Interventional, pre–post study design	USA	Academic healthcare system– primary care practice N = 2829	USPSTF criteria [55–80; ≥30; ≤15]
Goodley et al., 2023 [38]	Interventional, pre–post study design	UK	Primary care practices N = 10,708	[55–80; ever smoked] PLCOm2012NoRace
Jani et al., 2023 [39]	Retrospective	UK	Development cohort Primary care records N = 574,196 Validation cohort UK Biobank N = 137,918	[55–75]
Kukhareva et al., 2023 [40]	Interventional, pre–post study design	USA	30 primary care and 4 pulmonary clinics N = 1090	USPSTF 2013 guideline criteria
Liu et al., 2023 [41]	Retrospective	USA	Primary care clinic N = 102,475	2021 USPSTF guideline [50–80; ≥20; ≤ 15)
O’Brien et al., 2017 [42]	Mixed method pilot comparative study	Canada	Primary care practices N = 831	[55–74]
Ostrowski et al., 2021 [43]	Retrospective	Poland	Community N = 6631	Lung Cancer Screening NCCN Clinical Practice Guidelines ([50–79; ≥30]
Park et al., 2021 [44]	Retrospective	Korea	National Health Insurance Service N = 969,351 Patients	[40–79; ever-smokers]
Percac-Lima et al., 2018 [45]	RCT	USA	Five community health centers affiliated with an academic primary care network N = 1200	[55–77; current smokers]
Reuland et al., 2018 [46]	Interventional, pre–post study design	USA	Academic primary care practice N = 50	USPSTF criteria [55–80; ≥30; ≤15]
Schapira et al., 2023 [47]	RCT	USA	Veteran Affairs Medical Centers N = 140	[55–80; ≥30; ≤15]

CMC: Centers for Medicare and Medicaid Services; LLP: Liverpool Lung Project, including age, sex and smoking, family history of lung cancer, occupational exposure to asbestos, prior diagnosis of pneumonia, and prior diagnosis of a malignant tumor other than lung cancer; PLCOm2012: US Prostate, Lung, Colorectal, and Ovarian Cancer Screening Trial consisting of four smoking variables (smoking intensity, smoking duration, quit time in former smokers, and current smoking status [current versus former]) and seven non-smoking variables (age, race/ethnicity, socioeconomic circumstance estimated by education level, body mass index, personal history of cancer, chronic obstructive pulmonary disease, family history of lung cancer); py: pack-years; RCT: randomized controlled trial, UK: United kingdom, USA: United States of America, USPSTF United States Preventive Services Taskforce, y: years.

**Table 2:** Main findings of the studies.

Author/Year [Ref]	Screening Method	Pathway	Lung Cancer Diagnosis	Recruitment/ Follow-Up Period	Main Results
Azubuike et al., 2020 [30]	LDCT	QI project to increase provider compliance with the LCS guidelines. HCPs received education on guidelines on a new phone script for identifying at-risk patients from EHRs.	3 suspicious granulomas found, sent for additional follow-up	2017–2020/ 1 year	8/27 agreed to LDCT. The 19 patients who declined had concerns about testing, including radiation exposure, psychological distress, the efforts required to obtain the test, inability to take time off work, and lack of transportation. A significant difference in the number of LDCTs ordered from the preintervention (n = 0) to postintervention (n = 8) periods (*p* = 0.0043).
Brenner et al., 2018 [31]	LDCT	QI project to address three key quality gaps (EHR complete smoking history, VBR, SDM). Software-incorporated reminder system.	N/A	2015–2016/ 1 year	Percentage of completed smoking histories increased (22% pre-test to 47% post-test). Providers interacted with 27% of VBRs (172/644). Training decreased the frequency of deferral (16% pre-training vs. 7% post-training) and increased interaction with other features of the VBR (11% pre-training vs. 19% post-training).
Chiarantano et al., 2022 [33]	LDCT	Eligible participants referred from primary care or from screening campaign held on the “World No Tobacco Day”. Mobile Unit for screening. Trained primary care HCPs. Additional smoking cessation counseling and treatment.	3/233, diagnosis rate of 12.8/1000.	2019–2021/ 1 year	Participation in a smoking cessation group increased the odds of quitting smoking 2-fold (OR 2.16, CI 95%: 0.83–5.64, *p* value = 0.158). Less than 10% of the total high-risk population estimates were recruited.
Colamonici et al., 2023 [32]	LDCT	PCP-based, socially equitable, hybrid QI project on LCS in high-risk patients that incorporates patient education, SDM, and real-time tracking of the screening process.	Lung-RADS scores 4B/4X were more than double the expected prevalence (*p* = 0.008).	2021–2022/ 60 weeks	Increase in weekly LCS referrals from PCPs. Out of the 341 referrals, 229 scans were completed and scored.
Crosbie et al., 2022 [34]	LDCT	Eligible individuals from primary care records randomized to invitation to telephone LCS (intervention) or usual care. If eligibility criteria were met, a LHC appointment and baseline LDCT scan were offered through a mobile scanner. Smoking cessation advice offered during LHC.	N/A	2018–2021/ N/A	50.8% response rate in the intervention group. Of those responding, 34.4% were potentially eligible for screening, 29.9% attended a LHC, and 29.1% underwent LDCT screening. Responding reduced by 56% in people who currently smoked (adjusted OR 0.44, 95% CI 0.42–0.47). A similar pattern was seen for high socioeconomic deprivation, with response 42% lower in the most deprived IMD quintile compared with the least deprived (adjusted OR 0.58, 95% CI 0.54–0.62) and LHC attendance 22% lower (adjusted OR 0.78, 95% CI 0.62–0.98).
Currier et al., 2022 [35]	LDCT	PCPs assessed patient eligibility using EHRs and through care appointments, conducted SDM conversations with their patients about LDCT screening before referring them for LCS and support follow-up care after screening, including smoking cessation support. SDM education and resources provided to PCPs. Community stakeholders engagement in the screening program’s design and implementation.	2.11% (12/567)	2018–2020/ 3 years	In 2020, the LDCT lung cancer screening program successfully screened 6.9% of eligible adults compared to 0.93% in 2018. Adherence to follow-up scans increased from 51% in 2019 to 60% in 2020.
DiCarlo et al., 2022 [36]	LDCT	Participants identified through HER and randomized to Outreach Contact plus Decision Counseling (OC-DC,), Outreach Contact alone (OC), or usual care (UC). Participants in both the OC and OC-DC groups were mailed a decision aid (Option Grid™) including LCS educational materials. Within 10 days after the mailing, a study care coordinator attempted to make telephone contact with participants in the OC and OC-DC groups and assessed LCS eligibility, and for those who were eligible for screening discussed LCS. With OC-DC group participants, the care coordinator used an online interactive decision support software application (DCP) to guide participants through a brief decision-counseling session focused on eliciting values and clarifying preferences related to LCS. At the end of the session, the care coordinator used the application to compute an LCS preference score.	N/A	2019/ 90–280 days	LCS was significantly higher in the combined OC/OC-DC group versus UC controls (5.5% vs. 1.8%; HR = 3.28; 95%, CI: 1.98 to 5.41; *p* = 0.001). LCS was higher in the OC-DC group than in the OC group, although not significantly so (7% vs. 4%, respectively; HR = 1.75; 95% CI: 0.86 to 3.55; *p* = 0.123). LCS referral/scheduling was also significantly higher in the OC/OC-DC group compared to controls (11% vs. 5%; OR = 2.02; *p* = 0.001).
Fagan et al., 2020 [37]	LDCT	Eligible participants identified through her. Decision Counseling Program© (DCP) software used to guide a telephone-based SDM led by a trained decision counselor. Tobacco cessation hotline offered to all current smokers.	N/A	N/A/ 90 days	297/829 individuals were reachable by telephone, out of which 54 were eligible for screening with LDCT. 28 participants were recruited to the study, of which 20 completed SDM. 9 participants completed DCP and LDCT.
Goodley et al., 2023 [38]	LDCT	Population-based invitation approach. Letters were sent to all individuals from primary care records, inviting ever-smokers to attend an LHC. Attendees at higher risk (PLCOm2012NoRace score ≥ 1.5%) were offered two rounds of annual LDCT.	3.2% (144/4468)	2019–2020/ 2 years	83% of eligible respondents attended an LHC (n = 8887/10 708). Just over half of LHC attendees were eligible for screening (51%, n = 4540/8887), 98% of whom had a baseline LDCT scan (n = 4468/4540). Out of 4199 participants eligible for the second round, 83% (n = 3488) attended.
Jani et al., 2023 [39]	LDCT	Development and validation of an EHR-based lung cancer risk score (ALIGNED) from two large community cohorts. The new score was based on demographic information, smoking status, BMI, family history of lung cancer, and the presence of the following LTCs: alcohol misuse, COPD, coronary heart disease, dementia, hypertension, painful condition, stroke/TIA, peripheral vascular disease, and history of previous cancer, and previous pneumonia.	Six-year lung cancer incidence was 1.1% (6430) in the development and 0.48% (656) in the validation cohort.	2011–2017/ 6 years	The final model included 17/56 variables for the EHR-derived score: age, sex, socioeconomic status, smoking status, family history, BMI, BMI/smoking interaction, alcohol misuse, chronic obstructive pulmonary disease, coronary heart disease, dementia, hypertension, painful conditions, stroke, peripheral vascular disease, and history of previous cancer and previous pneumonia. The EHR-derived score had an AUC of 80.4% in the development and 74.4% in validation cohort and outperformed ever-smoked criteria.
Kukhareva et al., 2023 [40]	LDCT	Clinician-facing EHR prompts and an EHR-integrated everyday SDM tool designed to support the routine incorporation of SDM into primary care.	N/A	2019–2021/ 9 months	LDCT ordering and completion increased from 7.1% to 27.3% (*p* < 0.001) and from 4.4% to 17.7% (*p* < 0.001), respectively. A fivefold increase in the odds of LDCT scan imaging ordering for eligible patients.
Liu et al., 2023 [41]	N/A	Development and test of an NLP-based approach to extract smoking information from clinical notes to identify LCS eligible patients.	N/A	2019–2022/ 3 years	After adding NLP-extracted smoking information from clinical notes in 1 year and 3 years, the number of identified LCS-eligible patients were 8931 (51.7% increment) and 10,231 (73.8% increment), respectively NLP-based approach identified 119% more Black/African Americans who meet screening guidelines.
O’Brien et al., 2017 [42]	LDCT	All patients completed a pre-consultation questionnaire including questions of LCS. Practices were allocated to a screening electronic form (e-form) completion via pre-consultation software group or a paper form (*p*-form) group (completion of paper forms in the waiting room). After completing the screening form, patients in both e-form and *p*-form groups were invited to participate in a brief semi-structured telephone interview about their experience. Staff members were asked about their experiences implementing the screening forms, the impact on clinical functioning, and their interactions with patients.	N/A	2015/ 16 weeks	The number of patients who would be potentially eligible for LDCT screening based on their smoking history as assessed by patient responses was 116/831 (14%) overall, with 74/573 (13%) in the e-form group and 42/258 (16%) in the *p*-form group. Patients were willing to discuss lung cancer screening eligibility with their PCP.
Ostrowski et al., 2021 [43]	LDCT	Role of prediction models (I) Tammemagi’s PLCOm2012, (II) LLP, and (III) Bach’s lung cancer risk on risk assessment from the MOLTEST BIS program. Each participant underwent an LDCT, and selected participants underwent a further diagnostic work-up.	Lung cancer detection rate was 2.3%.	2016–2018/ 6 years	Based on the risk estimates by PLCOm2012, LLP, and Bach’s models, there were 82.4%, 50.3%, and 19.8% of the MOLTEST BIS participants, respectively, who fulfilled the above-mentioned threshold criteria of a lung cancer development probability. Of those detected for increased lung cancer risk, 97.4%, 74.0%, and 44.8% were eligible for screening by PLCOm2012, LLP, and Bach’s model criteria, respectively. In Tammemagi’s risk prediction model, only four cases (2.6%) would have been missed from the group of 154 lung cancer patients primarily detected in the MOLTEST BIS. All three models perform better than a screening program based on age and pack-years.
Park et al., 2021 [44]	LDCT	Role of prediction models for risk assessment with five models, including Bach, lung cancer risk models for screening (LCRAT), the Prostate, Lung, Colorectal, and Ovarian Cancer Screening Trial Model 2012 (PLCOM2012), Pittsburgh, and Liverpool Lung Project models (LLPi).	7.767/678.407 (1.14%) developed lung cancer in the training dataset. In the validation dataset 3368/290,994 (1.16%) developed lung cancer.	2007–2008/ 6.6 years	Models developed for ever-smokers in the Western population were applied to the Korean population; they moderately discriminated people who would develop and those who would not develop lung cancer (AUC, 0.66–0.81). The efficiency of risk model-based selection for lung cancer screening is superior to that of fixed criteria-based selection.
Percac-Lima et al., 2018 [45]	Any chest CT	Prior to the study, the principal investigator provided an educational session about LCS to PCPs. EHR was used to identify eligible subjects. Participants were randomized to intervention (IG) or usual care group (CG). The intervention group received invitation materials by means of mail and a call from the patient navigator. Navigators contacted patients to determine LCS eligibility, introduce shared decision-making about screening, schedule appointments with primary care physicians (PCPs), and help overcome barriers to obtaining a screening and follow-up.	LC was diagnosed in 8 participants in IG (2%) and 4 in CG (0.5%).	2016–2017/ 1 year	Percentage uptake of LDCT was 23.5% in the IG and 8.6% in the CG. Greater proportion of patients in the IG had any chest CT compared to patients in the CG (31% [124] vs. 17.3% [138], *p* < 0.001). LC screening CTs were performed in 94 IG patients (23.5%) vs. 69 CG (8.6%, *p* < 0.001). 20% of screened patients required follow-up.
Reuland et al., 2018 [46]	LDCT	Eligible patients identified by EHR were sent recruitment packages by post. Eligibility for LCS was determined by means of telephone, and patient was scheduled for an in-person visit. Study participants viewed the video at the clinic and completed a baseline knowledge survey, follow-up survey, and another survey at 3 mo.	Among the 10 completed LDCTs, 7 were Lung-RADS category 1 (normal result) and 2 were category 2 (small nodules, benign appearance). One was category 4a (suspicious findings); a 3-month follow-up scan showed resolution of the nodule.	2015–2017/ 3 months	36/50 participants had a clinic visit in the 3 months following study enrolment. Most participants (n = 48.96%) reported that the decision aid was “useful in making a decision about getting screened for lung cancer.” Knowledge increased from pre- to post-decision aid viewing (mean 2.6 vs. 5.5). 13/50 participants had an LDCT ordered. 10/50 participants completed an LDCT.
Schapira et al., 2023 [47]	LDCT	Participants eligible for LCS who had an upcoming appointment within 3 weeks were randomized to the LCSDecTool or control program. The LCSDecTool was designed to be used independently by the patient before the clinic visit, with the option to share some components with the clinician during the clinic visit. Smoking cessation support was also included in the tool.	N/A	2019–2021/ 3 years	Mean decisional conflict score at 1 month did not differ between the LCSDecTool and control groups (25.7 [95% CI, 21.4–30.1] vs. 29.9 [95% CI, 25.6–34.2], respectively; *p* = 0.18). Mean LCS knowledge score was greater in the LCSDecTool group immediately after intervention (7.0 [95% CI, 6.3–7.7] vs. 4.9 [95% CI, 4.3–5.5]; *p* < 0.001) and remained higher at 1 month (6.3 [95% CI, 5.7–6.8] vs. 5.2 [95% CI, 4.5–5.8]; *p* = 0.03) and 3 months (6.2 [95% CI, 5.6–6.8] vs. 5.1 [95% CI, 4.4–5.8]; *p* = 0.01). Uptake of LCS was greater in the LCSDecTool group at 6 months (26 of 69 [37.7%] vs. 15 of 71 [21.1%]; *p* = 0.04).

AUC: Area Under the Curve, CI: Confidence Interval, CT: computed tomography, DCP: decision counseling program, EHR: electronic health record, HCPs: healthcare professionals, HR: Hazard Ratio, LCT: lung cancer screening, LDCT: low-dose computed tomography, LHC: Lung Health Check, LTC: long-term conditions, N/A: Not Applicable, NPL: natural language processing, OR: Odds Ratio, PCP: primary care provider, QI: quality improvement, SDM: shared decision-making, VBR: Visit-Based Reminder.
